# Impact of the Naples Prognostic Score at admission on long-term prognosis among patients with coronary artery disease

**DOI:** 10.3389/fimmu.2025.1529779

**Published:** 2025-06-11

**Authors:** Bo Wang, Wan Chen, Lei Shi, Mingyu Pei, Yao Zhou, Yanlin Wei, Yutao Tang, Guozheng Qiu, Wenlong Duan, Shengxin Chen, Xiangrong Chen, Zhongyuan Zhang, Ying Shi, Qingwei Ji, Liwen Lyu

**Affiliations:** ^1^ Department of Emergency Medicine, Research Center of Cardiovascular Disease, The People’s Hospital of Guangxi Zhuang Autonomous Region, Guangxi Academy of Medical Sciences, Nanning, China; ^2^ Department of Cardiology, Research Center of Cardiovascular Disease, The People’s Hospital of Guangxi Zhuang Autonomous Region, Guangxi Academy of Medical Sciences, Nanning, China

**Keywords:** coronary artery disease, Naples Prognostic Score, long-term prognosis, mortality, inflammatory and nutritional status

## Abstract

**Background:**

The Naples Prognostic Score (NPS) is innovatively constructed to comprehensively evaluate the inflammatory and nutritional status according to several basic blood examinations. This study aimed to investigate the correlation between NPS and long-term prognosis in patients with coronary artery disease (CAD).

**Methods:**

The analysis data of this retrospective cohort study were collected from electronic health records in the People’s Hospital of Guangxi Zhuang Autonomous Region. All adult patients who underwent coronary angiology (CAG) and were diagnosed as having CAD at the People’s Hospital of Guangxi Zhuang Autonomous Region from March 2013 to December 2023 were enrolled. The primary endpoint was all-cause death during follow-up.

**Results:**

The 28,799 patients were divided into three groups according to the NPS value, with 803 (2.79%) in group 0, 12,130 (42.12%) in group 1, and 15,866 (55.09%) in group 2. Over the median follow-up period of 6.12 years, 3,630 patients (12.60%) died. Long-term all-cause mortality was significantly higher in group 2 and group 1 compared with group 0 (log-rank *p* < 0.001). Cox regression analysis showed that both continuous NPS and categorical NPS groups were significantly associated with the risk of all-cause mortality in patients with CAD [per 1-point decrement: full adjusted HR = 1.15; 95% CI, 1.11–1.19; compared with group 0 (NPS of 0), group 1 (NPS of 1 or 2), full adjusted HR = 1.38, 95% CI: 1.03–1.85, and group 2 (NPS of 3 or 4), full adjusted HR = 1.70, 95% CI: 1.27–2.28]. Restricted cubic spline analyses showed a linear relationship between NPS and risk of long-term all-cause death.

**Conclusions:**

The present study demonstrates that the NPS was independently associated with long-term all-cause mortality among patients with CAD.

## Introduction

The high prevalence and mortality rate caused by coronary artery disease (CAD) pose a serious public health challenge worldwide (1, 2). The identification of modifiable risk factors is crucial for implementing interventions on these variables to lower the risk of poor long-term prognosis. Previous studies reported that malnutrition and high inflammation status played important roles in the poor prognosis of patients with CAD (3–5). The immune and nutritional status was increasingly determined as a substantial prognostic risk factor in patients with CAD, independent of traditional CAD risk factors (6), whereas most validated predictors were just solitary inflammatory or nutrition-related markers, which caused the evaluations to become incomprehensive.

The Naples Prognostic Score (NPS) is innovatively constructed to comprehensively evaluate the inflammatory and nutritional status according to several basic blood examinations. It has been demonstrated that NPS is correlated to prognosis in various diseases (7–9). However, no studies verified the association between NPS and long-term outcomes in patients with CAD.

Accordingly, this research intended to explore the prognostic significance of baseline NPS at admission, aiming to offer a simple and reliable approach to identify high-risk individuals among the CAD population.

## Methods

### Study design and data collection

A total of 39,653 individuals who underwent coronary angiography (CAG) for proven CAD at the People’s Hospital of Guangxi Zhuang Autonomous Region during March 2013 and December 2023 were enrolled. This research adopted a retrospective cohort design. A retrospective cohort design was used in this study. The following clinical characteristics were collected from electronic health records (EHRs): demographic characteristics, laboratory examination, and medication at discharge. The final database was cross-validated across multiple sources (e.g., laboratory systems and billing records) to minimize inconsistencies. Follow-up information was obtained and preserved by trained researchers. The study was approved by the Ethics Committee of Guangxi Zhuang Autonomous Region People’s Hospital and conducted following the Declaration of Helsinki (IRB No. KY-QT-202103).

**Figure 1:**
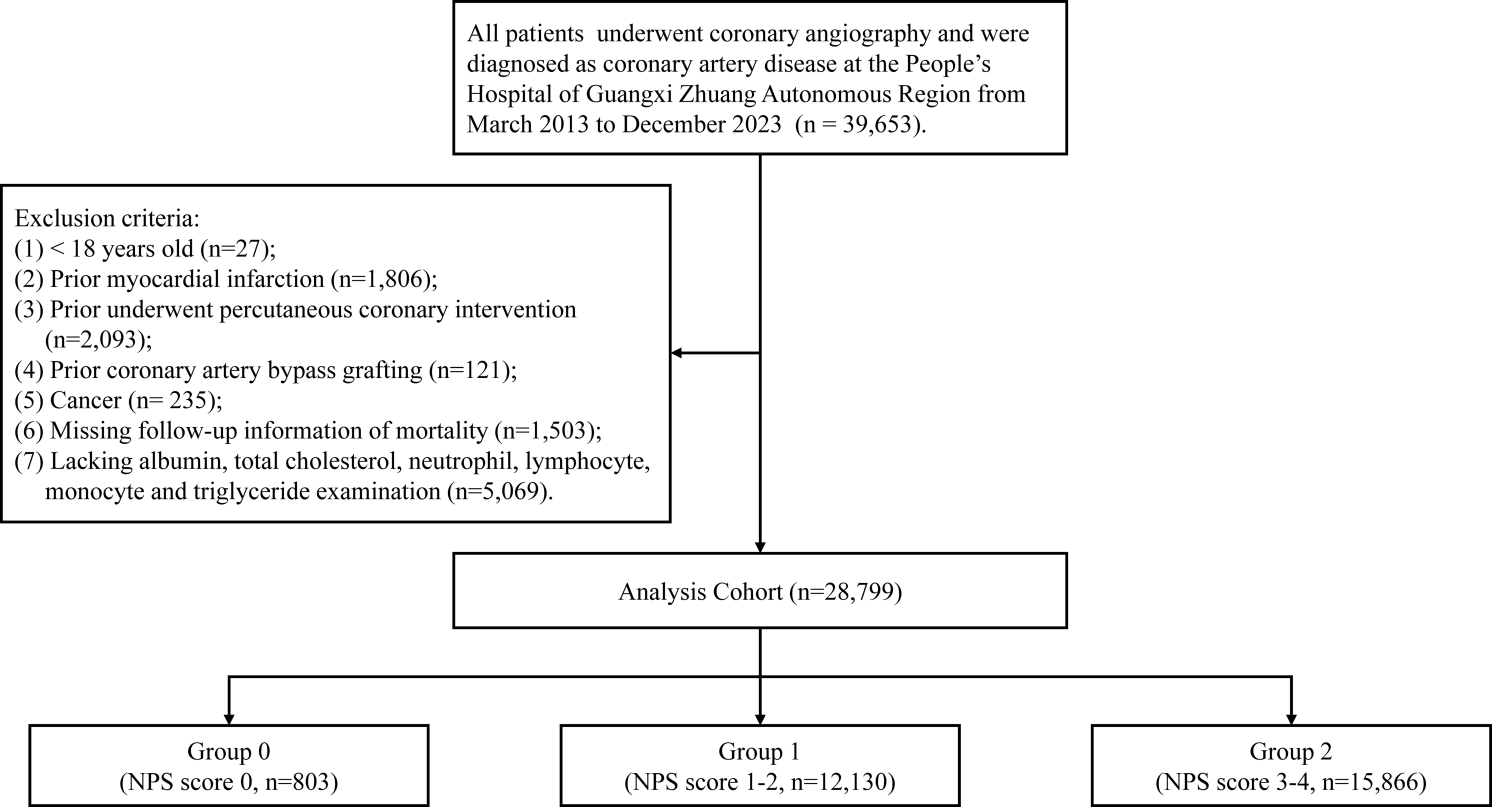
Study flowchart.

Patients fulfilling the following criteria were excluded: (1) individuals aged less than 18 years; (2) individuals with a history of myocardial infarction; (3) individuals who had undergone percutaneous coronary intervention (PCI) previously; (4) individuals who had undergone coronary artery bypass grafting previously; (5) individuals diagnosed with cancer; (6) individuals lacking follow-up information; and (7) individuals lacking albumin, total cholesterol, neutrophil, lymphocyte, monocyte, or triglyceride examination results. **Figure 1** shows the flowchart of this study.

### Clinical definitions

The primary outcome of this study was the all-cause mortality. The diagnosis of CAD was derived from angiographic confirmation (>50% stenosis in one vessel at the lowest), which was derived from structured EHR data (e.g., procedural reports and cardiologist notes). To assess NPS value, the following four variables were applied: serum albumin concentrations, total cholesterol concentrations, neutrophil-to-lymphocyte ratio (NLR) levels, and lymphocyte-to-monocyte ratio (LMR) levels (6). According to previous reports, serum albumin ≥40 g/L, total cholesterol > 180 mg/dL (2.03 mmol/L), LMR > 4.44, or NLR ≤ 2.96 was scored as 0, while serum albumin <40 g/L, total cholesterol ≤ 180– mg/dL (2.03 mmol/L), LMR ≤ 4.44, or NLR > 2.96 was scored as 1. NPS is the sum of the scores of each of the four factors. To convert total cholesterol from mmol/L to mg/dL, multiply by 88.6. Details were reported in **Table 1**. All procedures for PCI as well as CAG followed the clinical standard guidelines (10–12). A variety of concomitant diseases were taken into consideration, including acute myocardial infarction (AMI), congestive heart failure (CHF), hypertension, diabetes mellitus, chronic kidney disease (CKD), anemia, chronic obstructive pulmonary disease (COPD), and stroke. CHF was defined as New York Heart Association (NYHA) class >2 or Killip class >1 (13). CKD was determined as estimated glomerular filtration rate (eGFR) less than 60 mL/min/1.73 m^2^ (14). The level of eGFR was calculated based on the Modification of Diet in Renal Disease (MDRD) formula (15). Other comorbidities were derived from ICD codes plus clinician diagnosis.

**Table 1:** Calculation of the Naples Prognostic Score (NPS).

Components	Cutoff value	Points
Albumin	≥40 g/L	0
	<40 g/L	1
Total cholesterol	>180 mg/dL (2.03 mmol/L)	0
	≤180 mg/dL (2.03 mmol/L)	1
LMR	>4.44	0
	≤4.44	1
NLR	>2.96	1
	≤2.96	0

LMR, lymphocyte-to-monocyte ratio; NLR, neutrophil-to-lymphocyte ratio.

### Statistical analysis

Based on the final score, study population was categorized into 3 groups (group 0: NPS value of 0; group 1: NPS value of 1 or 2; group 2: NPS value of 3 or 4) (6). This research compared continuous variables using the Wilcoxon rank sum test or Student’s *t* test and expressed them as mean with median with interquartile range (IQR) or standard deviation (SD). For categorical variables, the chi-square test was applied and number and percentage (%) were reported. The Kaplan–Meier curves were applied to estimate the effect of different groups on long-term all-cause mortality. To determine and quantify the magnitude of risk, univariate and multivariate COX regression were conducted. The study carefully selected covariates to create four models: (1) univariate; (2) adjusted age and gender; (3) adjusted age, gender, PCI and morbidities; and (4) adjusted all covariates above as well as medication use of renin–angiotensin system inhibitor (RASi), statins, and β-blocker. The same covariates were used in restricted cubic spline (RCS) analyses to investigate potential nonlinear correlations. R software (version 3.6.3) was used for all statistical analyses. A *p*-value of less than 0.05 was considered statistically significant.

## Results

### Patient characteristics

Study participants were 28,799 patients (mean age 63.10 ± 10.56 years, 74.75% men) suffering from CAD (**Table 2**). Of these patients, 803 (2.79%) were classified as group 0, 12,130 (42.12%) were classified as group 1, and 15,866 (55.09%) were classified as group 2. There were 21,148 (73.43%) patients who underwent PCI, 5,795 (20.12%) had AMI, 2,260 (7.86%) had CHF, 16,268 (56.49%) had hypertension, 7,595 (26.37%) had diabetes mellitus, 5,757 (20.89%) had CKD, 248 (0.86%) had COPD, and 1,568 (5.44%) had stroke.

**Table 2:** Baseline characteristics of the study population.

Characteristics*	Overall	Group 0	Group 1	Group 2	*P* value
	(*N* = 28,799)	(*N* = 803)	(*N* = 12,130)	(*N* = 15,866)	
Demographic					
Age, years	63.10 (10.56)	58.78 (9.66)	61.56 (10.31)	64.50 (10.57)	<0.001
Male, *n* (%)	21,526 (74.75)	468 (58.28)	8,376 (69.05)	12,682 (79.93)	<0.001
Medical history					
AMI, *n* (%)	5,795 (20.12)	35 (4.36)	1,422 (11.72)	4,338 (27.34)	<0.001
CHF, *n* (%)	2,260 (7.86)	29 (3.61)	639 (5.27)	1,592 (10.05)	<0.001
Hypertension, *n* (%)	16,268 (56.49)	428 (53.30)	6,631 (54.67)	9,209 (58.04)	<0.001
Diabetes mellitus, *n* (%)	7,595 (26.37)	210 (26.15)	3,122 (25.74)	4,263 (26.87)	0.10
CKD, *n* (%)	5,757 (20.89)	81 (10.64)	1,853 (16.08)	3,823 (25.03)	<0.001
COPD, *n* (%)	248 (0.86)	3 (0.37)	77 (0.63)	168 (1.06)	<0.001
Stroke, *n* (%)	1,568 (5.44)	18 (2.24)	511 (4.21)	1,039 (6.55)	<0.001
PCI, *n* (%)	21,148 (73.43)	525 (65.38)	8,565 (70.61)	12,058 (76.00)	<0.001
Laboratory examination					
Albumin, g/L	36.05 (4.04)	42.19 (1.79)	37.46 (3.69)	34.66 (3.69)	<0.001
Total cholesterol, mmol/L	4.54 (1.09)	5.75 (0.80)	4.99 (1.06)	4.13 (0.94)	<0.001
HDL-C, mmol/L	1.00 (0.26)	1.16 (0.28)	1.05 (0.27)	0.96 (0.25)	<0.001
LDL-C, mmol/L	2.77 (0.92)	3.57 (0.85)	3.09 (0.93)	2.49 (0.80)	<0.001
Triglyceride, mmol/L	1.63 (1.08)	2.29 (1.78)	1.82 (1.22)	1.44 (0.85)	<0.001
Neutrophil, 109/L	4.99 (2.06)	3.93 (1.12)	4.24 (1.48)	5.62 (2.26)	<0.001
Lymphocyte, 109/L	1.93 (0.66)	2.54 (0.63)	2.22 (0.63)	1.68 (0.57)	<0.001
Monocyte, 109/L	0.62 (0.23)	0.46 (0.14)	0.56 (0.20)	0.68 (0.23)	<0.001
NLR	2.99 (1.97)	1.59 (0.49)	2.02 (0.98)	3.79 (2.20)	<0.001
LMR	3.44 (1.70)	5.77 (1.67)	4.34 (1.81)	2.64 (1.04)	<0.001
Medication					
RASi, *n* (%)	14,417 (50.86)	330 (41.30)	5,978 (49.84)	8,109 (52.15)	<0.001
β-blocker, *n* (%)	22,812 (80.48)	639 (79.97)	9,628 (80.27)	12,545 (80.68)	0.65
Statin, *n* (%)	26,814 (94.60)	753 (94.24)	11,306 (94.26)	14,755 (94.89)	0.06
Clinical outcome					
All-cause mortality	3,630 (12.60)	51 (6.35)	1,218 (10.04)	2,361 (14.88)	<0.001

AMI, acute myocardial infarction; CHF, congestive heart failure; CKD, chronic kidney disease; COPD, chronic obstructive pulmonary disease; PCI, percutaneous coronary intervention; HDL-C, high-density lipoprotein cholesterol; LDL-C, low-density lipoprotein cholesterol; NLR, neutrophil-to-lymphocyte ratio; LMR, lymphocyte-to-monocyte ratio; RASi, renin–angiotensin system inhibitor.

*Data are presented as the mean value (standard deviation) or number of participants (percentage).

Patients in groups 2 and 1 were older on average compared to group 0. Moreover, the prevalence of comorbid conditions such as AMI, CHF, hypertension, CKD, COPD, and stroke was also higher in group 2 and group 1 than in group 0. For laboratory examination, the concentrations of albumin, total cholesterol, HDL-C, LDL-C, triglyceride, lymphocyte, and LMR were lower in groups 2 and 1. Meanwhile, neutrophil, monocyte, and NLR were higher in groups 2 and 1.

### Primary outcomes

**Figure 2:**
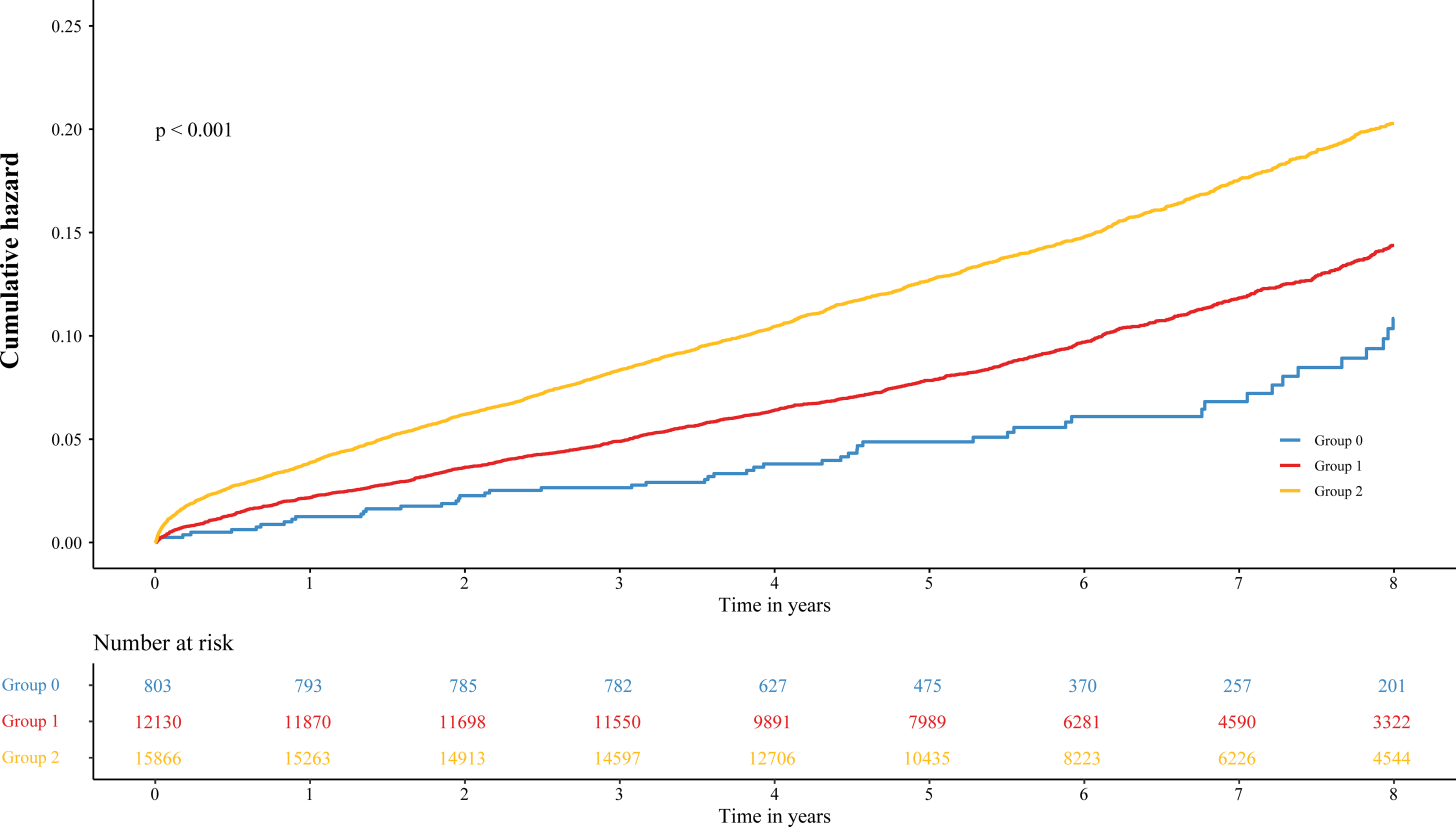
Cumulative incidence of all-cause death for three NPS groups.

During the median follow-up period of 6.12 years, 3,630 patients (12.60%) died. Of these, 51 (6.35%), 1,218 (10.04%), and 2,361 (14.88%) were in groups 0, 1, and 2, respectively. On the basis of Kaplan–Meier cumulative hazard curves shown in **Figure 2**, a significantly higher cumulative incidence was determined from group 2 (log-rank *p* < 0.001) versus group 0 and group 1. In **Table 3**, results of Cox regression are shown. When NPS value was analyzed as a continuous variable, per 1-point increasement was related to 15% increased risk (adjusted HR, 1.15; 95% CI, 1.11–1.19). When NPS value was analyzed as a categorical variable, patients in group 2 (NPS of 3 or 4) and group 1 (NPS of 1 or 2) have a 70% and 38% increased risk compared to group 0 (NPS of 0) patients with low levels.

**Table 3:** Cox proportion hazard model stratified by NPS group for all-cause mortality.

Model	Model 1		Model 2		Model 3		Model 4	
	HR (95% CI)	*p*-value	HR (95% CI)	*p*-value	HR (95% CI)	*p*-value	HR (95% CI)	*p*-value
NPS was analyzed as a continuous variable								
Per 1-point increment	1.29 (1.24–1.33)	<0.001	1.21 (1.16–1.25)	<0.001	1.16 (1.11–1.20)	<0.001	1.15 (1.11–1.19)	<0.001
NPS was analyzed as a categorical variable								
Group 1 (0)	Ref	–	Ref	–	Ref	–	Ref	–
Group 2 (1–2)	1.53 (1.16–2.03)	0.003	1.37 (1.03–1.81)	0.03	1.34 (1.01–1.79)	0.04	1.38 (1.03–1.85)	0.03
Group 3 (3–4)	2.30 (1.74–3.03)	<0.001	1.84 (1.39–2.43)	<0.001	1.68 (1.26–2.25)	<0.001	1.70 (1.27–2.28)	<0.001

NPS, Naples Prognostic Score.

Model 1: unadjusted.

Model 2: adjusted age and gender.

Model 3: adjusted age, gender, PCI, and morbidities including AMI, CHF, hypertension, diabetes mellitus, CKD, COPD, and stroke.

Model 4: adjusted all covariates above and medication use of RASi, statins, and β-blocker.

**Figure 3:**
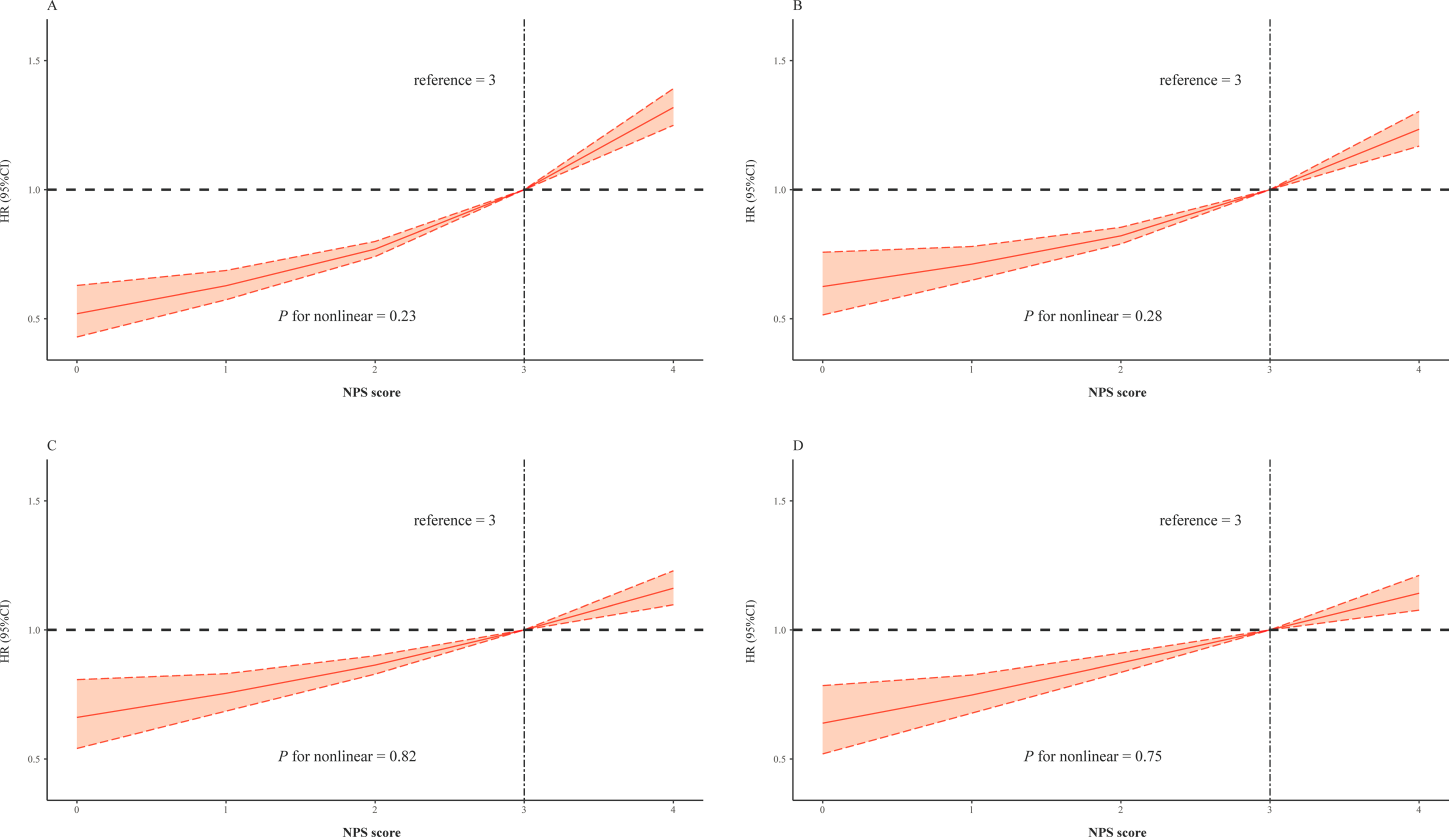
Restricted cubic splines of the NPS and hazard ratio for mortality. **(A)** The restrict spline curve of the univariate Cox model. **(B)** The restrict spline curve of multivariate Cox model 2, adjusted age and gender. **(C)** The restrict spline curve of multivariate Cox model 3, adjusted age, gender, PCI, and morbidities including AMI, CHF, hypertension, diabetes mellitus, CKD, COPD, and stroke. **(D)** The restrict spline curve of multivariate Cox model 4, adjusted all covariates above and medication use of RASi, statins, and β-blocker.

In order to examine the existing potential nonlinear correlation, RCS analysis was conducted with the same covariates form four Cox models (**Figure 3**). The result illustrated positive linear correlation between NPS and risk of outcome (all *p* for nonlinear > 0.05, **Figure 3**).

### Subgroup analysis

**Figure 4:**
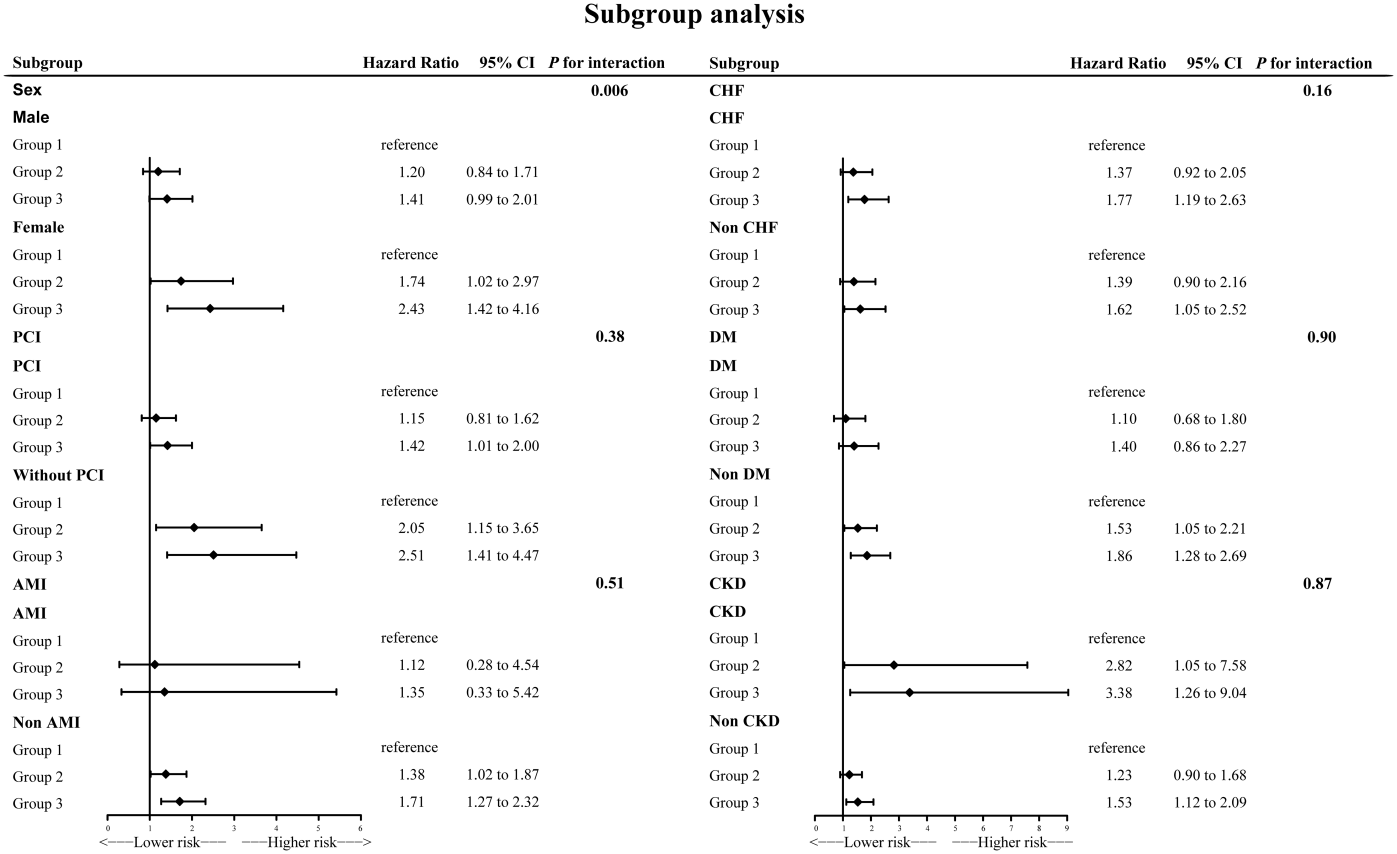
Subgroup analysis.

Subgroup analyses generally agreed with main analysis results (**Figure 4**). There was a significant interaction effect for gender (*p* for interaction = 0.006). Among female individuals, the results were even more dramatic.

## Discussion

This cohort study of participants with CAD examined the association between NPS value and risk of all-cause death. The result clearly demonstrated a linear positive correlation between NPS and the risk of long-term all-cause death, with each point increase in NPS increasing the risk by 15% in CAD population. Additionally, patients in the high NPS value group had a poorer prognosis compared to the group with an NPS value of 0. After adjustment of all confounders, the results were still robust. According to further subgroup analysis, most results were consistent.

The NPS is a newly detected scoring system that can comprehensively evaluate the individual’s immunological and nutritional status according to several basic blood examinations. These easily accessible and routinely tested indicators included albumin level, total cholesterol concentration, lymphocyte, neutrophil, and monocyte. NPS was initially constructed and verified as an independent prognostic risk factor in colorectal cancer (6). Subsequently, the NPS’s ability to predict prognosis was validated in various diseases, and increased NPS levels were found to be independently correlated to increased risk of poor prognosis (7–9). Erdogan et al.’s study, which included 1,887 consecutive patients with STEMI who were undergoing PCI, reported that the high-NPS group had a higher rate of death compared to the low-NPS group (19.1% vs 7.8%, *p* < 0.001) (16). After adjusting confounders, evaluated NPS was associated with poorer prognosis and high NPS (3–4) increased the risk 1.49-fold. In Saygi et al.’s study that recruited 3,828 patients with STEMI with emergency PCI, the result illustrated that the rate of in-hospital death was elevated in the high-NPS group in contrast to the medium- and low-NPS group (17). Multivariable logistic regression also showed that the high- and medium-NPS group significantly increased the risk of in-hospital death.

NPS encompasses not only immunoinflammatory markers like NLR and LMR, but also total cholesterol and serum albumin. These indicators mirrored an individual’s nutritional status and inflammation level. As a result, the body condition of the patient could be evaluated in a more comprehensive and efficient manner. According to the NPS scoring system, high NPS levels most likely presented underlying low albumin, low total cholesterol, low LMR, and high NLR, suggesting malnutrition and high inflammation status. The significance of inflammation in initiating, promoting, and destabilizing atherosclerotic plaques is paramount (18, 19). Systemic inflammation was commonly related to vascular wall inflammation (20). Several clinical studies demonstrated that taking anti-inflammatory drugs, such as therapeutic monoclonal antibodies against IL-1β (canakinumab) and colchicine is useful in modulating inflammation, reducing adverse events risk and improving the prognosis of chronic CAD (21, 22). A growing body of research has suggested that inflammation-related indexes, such as LMR and NLR, are related to CAD severity and prognosis (23, 24). NLR and LMR are straightforward and economically efficient biomarkers that mirror the complex equilibrium between innate and adaptive immune responses (25).

Notably, NLR has been demonstrated to be a reliable biomarker of inflammation in the vascular wall, and it is readily accessible (26). Through reactive oxygen species, cytokines, proteases, and neutrophil extracellular traps, neutrophils were known to adversely affect chronic inflammatory disorders in individuals with increased NLR (27). LMR is another inflammatory indicator calculated based on lymphocytes and monocytes. Decreased LMR was related to the poor prognosis. Inflammatory cell infiltration is an important mechanism of atherosclerosis (20, 28). Lymphocytes and monocytes played crucial roles in atherosclerosis’ early stage; meanwhile, neutrophils were involved in plaque destabilization and thrombosis (29).

Furthermore, nutrition, being a modifiable element, had a further impact on the prognosis of patients with CAD. Malnutrition, in particular, significantly affects prognosis. Several studies have identified malnutrition as the most prevalent cause of secondary immunologic disorders. Low serum total cholesterol levels and albumin concentrations were important objective indicators of malnutrition. Previous research has demonstrated that a low total cholesterol level serves as a biological indicator for concurrent cachexia, malnutrition, cancer, and other chronic diseases, which have confirmed detrimental effects for prognosis (30). Additionally, there is emerging evidence suggesting that cholesterol levels were clearly associated with regulation of immune cell function. Decreased cholesterol levels result in diminished activation of immune signaling and reduced antitumor activity (31). Serum albumin concentrations were linked to both nutritional status and the acute phase reaction, as well as chronic inflammatory diseases (32, 33). Existing studies indicated that decreased albumin concentrations might serve as an indicator of sustained arterial injury and advancement of thrombosis as well as atherosclerosis (34). As a result of decreased albumin concentrations, catabolic cytokines were produced, muscle breakdown occurred, and appetite was suppressed (33). Given the established roles of neutrophils, monocytes, lymphocytes, serum albumin, and total cholesterol in the poor prognosis of patients with CAD, NPS emerges as a promising tool. NPS calculated by combining these factors not only is less susceptible to various non-pathological factors than individual indicators, but also captures the patient’s inflammatory and nutritional status, which are crucial in the prognosis of patients with CAD. Our study validated the association between NPS and poor prognosis in patients with CAD using large sample data, and found a linear correlation between NPS level and the risk of poor prognosis. This may make NPS a good prognostic assessment tool for patients with CAD. It enables clinicians to identify high-risk patients at an early stage. However, precisely because NPS is a comprehensive assessment of nutritional status and inflammation levels, timely clinical interventions targeting high-risk patients need to be further explored.

According to the results of subgroup analysis, the association between NPS and long-term all-cause death was more significant in female patients (*p* for interaction = 0.006). The value of HR was also higher in female patients. There may be several possible explanations. Firstly, women passed through adverse metabolic disturbances and lipid profile deterioration more than men (35, 36). A previous study conducted in humans and mice confirmed that estrogen preserves endothelial function (37, 38). Thus, because of older age at onset and decreased estrogen levels, endothelial dysfunction is more severe in female patients with CAD than in male patients. The above factors together resulted in a worse nutritional status and higher levels of inflammation played more important roles in female patients’ prognosis. Secondly, women tend to present later CAD than men and suffered more from chronic comorbidities. Several present studies including Steg et al.’s CLARIFY study and Chen et al.’s study demonstrated this (39, 40). Furthermore, the clinical presentation was more atypical in women, and female patients received fewer interventions and drug therapies (41, 42). NPS may provide additional clues apart from the control of comorbidities such as hypertension, CHF, and CKD to improve prognosis in female patients with CAD.

It is necessary to admit that there existed several certain limitations. Firstly, although this research was derived from a single-center retrospective cohort, the enrolled patients originated from the largest cardiac intervention center in the Guangxi Zhuang Autonomous Region, which rendered the sample representative and ensured the study’s quality control. Secondly, the generalization of our results is restricted to the Chinese population without taking into account other races. Thirdly, this study only evaluated the baseline admission NPS levels and did not assess the effect of NPS changes during follow-up. This should be further investigated. Fourthly, there were limited data on the included patients, without information about body mass index (BMI), smoking status, and socioeconomic factors. This may partially affect the results. However, we adjusted for potential confounders whenever possible and constructed three multivariate Cox regression models to ensure the robustness of the results.

## Conclusion

This research indicated the significance of inflammation and nutritional status in case of an unfavorable prognosis. Based on routine examination, NPS can comprehensively evaluate the prognosis of patients with CAD. There was a linear positive correlation between NPS value and prognosis.

## Data Availability

The datasets presented in this article are not readily available because it is not applicable at this stage. The datasets analyzed during the current study will be available from the corresponding author on reasonable request when the study is finished. Requests to access the datasets should be directed to LL iculvliwen@163.com.
